# International Congress

**Published:** 1889-09

**Authors:** 


WE HAVE PUBLISHED elsewhere the results of the International Congress for the consideration of questions relative to Alcoholism.

Especially worthy of thought are the resolutions adopted, to recommend that chronic alcoholics, guilty of a breach of law, shall be sent to houses of detention, whose object shall be rather to cure than to punish.

We can but wish that the United States had been represented at this Congress, and that there might be a chance that the ideas advanced could find root in this country.

